# Potential determinants of antibody responses after vaccination against SARS-CoV-2 in older persons: the Doetinchem Cohort Study

**DOI:** 10.1186/s12979-023-00382-4

**Published:** 2023-10-25

**Authors:** Yunus Kuijpers, H. Susan J. Picavet, Lia de Rond, Mary-lène de Zeeuw-Brouwer, Ryanne Rutkens, Esther Gijsbers, Irene Slits, Peter Engelfriet, Anne-Marie Buisman, W. M. Monique Verschuren

**Affiliations:** 1 Centre for Prevention, Lifestyle and Health, National Institute for Public Health and the Environment (RIVM), Bilthoven, 3721 MA The Netherlands; 2 Centre for Immunology of Infectious Diseases and Vaccines, National Institute for Public Health and the Environment (RIVM), Bilthoven, 3721 MA The Netherlands; 3 Julius Centre for Health Sciences and Primary Care, University Medical Centre Utrecht, Utrecht University, Utrecht, 3508 TC The Netherlands

**Keywords:** COVID-19 vaccination, Antibody responses, Age, Frailty, Comorbidity, Lifestyle deficits

## Abstract

**Background:**

Immune responses to vaccination vary widely between individuals. The aim of this study was to identify health-related variables potentially underlying the antibody responses to SARS-CoV-2 vaccination in older persons. We recruited participants in the long-running Doetinchem Cohort Study (DCS) who underwent vaccination as part of the national COVID-19 program, and measured antibody concentrations to SARS-CoV-2 Spike protein (S1) and Nucleoprotein (N) at baseline (T0), and a month after both the first vaccination (T1), and the second vaccination (T2). Associations between the antibody concentrations and demographic variables, including age, sex, socio-economic status (SES), comorbidities (cardiovascular diseases and immune mediated diseases), various health parameters (cardiometabolic markers, inflammation markers, kidney- and lung function) and a composite measure of frailty (‘frailty index’, ranging from 0 to 1) were tested using multivariate models.

**Results:**

We included 1457 persons aged 50 to 92 years old. Of these persons 1257 were infection naïve after their primary vaccination series. The majority (*N* = 954) of these individuals were vaccinated with two doses of BNT162b2 (Pfizer) and their data were used for further analysis. A higher frailty index was associated with lower anti-S1 antibody responses at T1 and T2 for both men (*R*_T1_ = -0.095, *P*_T1_ = 0.05; *R*_T2_ = -0.11, *P*_T2_ = 0.02) and women (*R*_T1_ = -0.24, *P*_T1_ < 0.01; *R*_T2_ = -0.15, *P*_T2_ < 0.01). After correcting for age and sex the frailty index was also associated with the relative increase in anti-S1 IgG concentrations between the two vaccinations (β = 1.6, *P* < 0.01). Within the construct of frailty, history of a cardiac catheterization, diabetes, gastrointestinal disease, a cognitive speed in the lowest decile of the population distribution, and impaired lung function were associated with lower antibody responses after both vaccinations.

**Conclusions:**

Components of frailty play a key role in the primary vaccination response to the BNT162b2 vaccine within an ageing population. Older persons with various comorbidities have a lowered immune response after their first vaccination, and while frail and sick older persons see a stronger increase after their second vaccination compared to healthy people, they still have a lower antibody response after their second vaccination.

**Supplementary Information:**

The online version contains supplementary material available at 10.1186/s12979-023-00382-4.

## Introduction

The COVID-19 pandemic was a global outbreak of disease caused by a novel coronavirus. The rapid development and implementation of SARS-CoV-2 vaccination programs helped reduce symptomatic COVID-19 and protect against severe COVID-19 in the general population [1, 2]. These programs included the use of two new mRNA-based vaccines, one of which was the BNT162b2 (Pfizer) vaccine which required two injections to complete the primary vaccination series.

With increasing age, physical functions decline. However, there is large heterogeneity in health at older ages [3, 4]. This heterogeneity in health status also extends to that in the immune system [5, 6]. The age-related decline in function of the immune system, called immunosenescence, affects and is affected by various autoimmune, cardiovascular, neurodegenerative, and infectious diseases as well as lifestyle and genetics [7]. In an earlier study, we have seen that after the initial COVID-19 vaccinations there is much heterogeneity in antibody responses amongst older persons [8]. There is, however, a lack of studies focusing on how general health influences the immune responses to vaccination against SARS-CoV-2 in the general population.

The response to SARS-CoV-2 vaccination itself has been extensively researched in the general population and in health care workers in various studies, but older persons tend to be underrepresented in such studies [9]. Further research has been done in specific patient subpopulations with diseases such as cancer, autoimmune disorders, (kidney) transplantations, and with other specific comorbidities [10–14]. These studies tend to focus on patients with diseases that affect the immune system or require immune modulating medication, but do not compare these groups directly to each other or to community dwelling (more healthy) older vaccinee’s from the general population. It is well known that humoral immune responses are lower in frail elderly. This applies also to responses to vaccination, including anti-SARS-CoV-2 vaccination [15]. However, while other studies have characterized the immune response after vaccination in a healthy and frail older population they did not investigate other potential determinants in addition to frailty status, or what aspects within the construct of frailty determined the vaccine response [16, 17]. Furthermore, the vast majority of frailty related research in this field uses the less comprehensive clinical frailty scale and not the frailty index.

In the current study we have used the long-running Doetinchem Cohort Study (DCS) [18, 19], a unique population based longitudinal cohort representative of the Dutch general population, that includes individuals now ranging from 50 to 92 years of age. In this cohort we aimed to study potential determinants of heterogeneity in antibody responses to the primary vaccination series with BNT162b2 (Pfizer), using an extensive set of characteristics of overall (physical and cognitive) health, including various comorbidities and other manifestations of frailty. By identifying which factors influence antibody responses upon primary vaccination we aimed to contribute towards the design of possibly more targeted vaccination strategies in the future.

## Results

### General characteristics

**Fig. 1:**
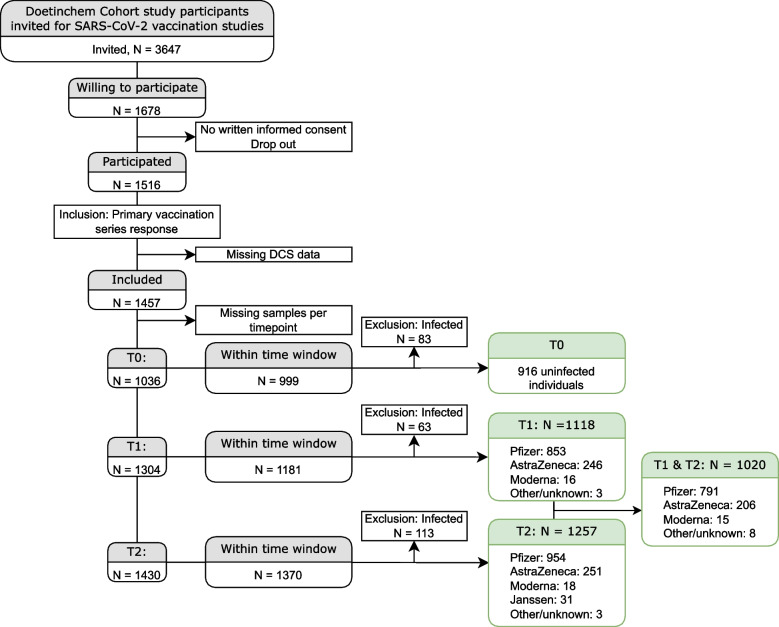
Flowchart of the study cohort. Participants were excluded in case of a missing signed informed consent, not sending samples (drop outs), or a missing sample at a month post 2^nd^ vaccination (T2). For further data analysis, samples taken outside the time window around the predefined timepoints post vaccination were excluded, participants already infected with SARS-CoV-2 before vaccination and participants missing data on co-morbidities and other health parameters since round 6 of the Doetinchem cohort study were also excluded

The baseline characteristics of our cohort, including all variables used in this study are shown in Table 1. Three thousand six hundred forty-seven individuals were contacted of which 1678 were willing to participate. One thousand five hundred sixteen persons provided written consent forms and did not drop out of the study; of these people, 149 individuals were infected with SARS-CoV-2 during the course of the study. One thousand four hundred fifty-seven individuals had antibody concentrations measured and were included in the study, 74.0% and 76.7% received the BNT162b2 vaccine for their first and second vaccination which can be seen in the flow diagram of Fig. 1. Included in the table is a summary measure of their frailty, the frailty index which ranges from 0 (non-frail) to 1 (maximum level of frailty). A more in-depth description of the various comorbidities as well as the different parameters that make up the frailty index can be seen in Table S1. In addition, we have compared the vaccination sub-cohort with the entire Doetinchem Cohort Study and beyond the socio-economic status and the fraction of people with multiple comorbidities the vaccination study subgroup resembles the entire study cohort as can be seen in Table S2.

**Table 1:** Prevalence and mean (SD) of sociodemographic, cardiometabolic, and comorbidity related variables in the study population

	T1: *N* = 853	T2: *N* = 954	T1 & T2: *N* = 791
**Sociodemographic**			
Women (%)	47.9	49.9	48.2
Age (years) (mean (SD))	69.1 (7.6)	69.3 (7.8)	69.3 (7.6)
Socio-Economic Status (%)			
*Low*	32	34	33
*Middle*	34	34	33
*High*	34	32	34
***Lifestyle***			
Current smokers (%)	6.7	6.5	7
Drinking alcohol (%)	71	69	70
Adherence to Dutch healthy exercise norm (NNGB) (%)	66	64	66
**Cardiometabolic factors**			
Waist circumference (cm) (mean (SD))	96.0 (11.9)	96.3 (12.0)	96.0 (12.0)
BMI (kg/m^2^) (mean (SD))	26.4 (4.1)	26.6 (4.2)	26.4 (4.1)
Systolic blood pressure (mmHg) (mean (SD))	133 (17)	132 (17)	133 (17)
Total cholesterol (mmol/L) (mean (SD))	5.3 (1.0)	5.3 (1.0)	5.3 (1.0)
HDL cholesterol (mmol/L) (mean (SD))	1.5 (0.4)	1.5 (0.4)	1.5 (0.4)
Creatinine (mmol/L) (mean (SD))	83.1 (16.4)	82.7 (16.6)	83.0 (16.5)
Glucose (mmol/L) (mean (SD))	5.8 (1.6)	5.8 (1.6)	5.8 (1.6)
GlycA (mmol/L) (mean (SD))	0.9 (0.1)	0.9 (0.1)	0.9 (0.1)
CRP (mmol/L) (mean (SD))	2.0 (4.2)	2.2 (4.3)	2.1 (4.3)
**Comorbidity related variables**			
Frailty index (median (IQR))	0.07 (0.03–0.11)	0.08 (0.03–0.11)	0.07 (0.03–0.11)
FEV1 max (mean (SD))	3.1 (0.8)	3.0 (0.8)	3.1 (0.8)
FVC max (mean (SD))	4.2 (1.0)	4.1 (1.0)	4.2 (1.1)
FEV1/FVC ratio (mean (SD))	0.7 (0.1)	0.7 (0.1)	0.7 (0.1)
eGFR (mean (SD))	0.9 (0.2)	0.9 (0.2)	0.9 (0.2)
Having > = 1 comorbidity (%)	62	60	61

### BNT162b2 induced antibody responses during the primary vaccination series

**Fig. 2:**
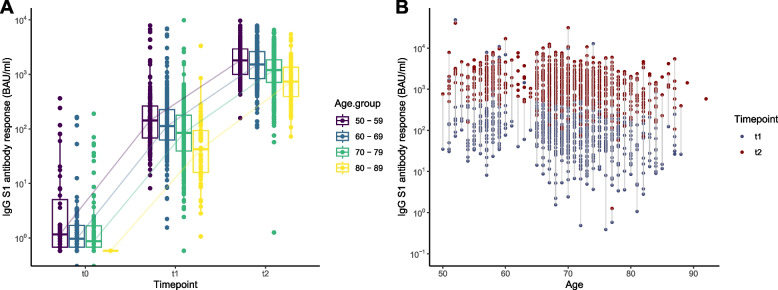
Distribution of the SARS-CoV-2 -S1 IgG antibody concentrations in binding antibody units per milliliter (BAU/ml) at T0 (before vaccination), T1 (1 month after first vaccination), and T2 (1 month after second vaccination) in persons above 50 years of age per ten year age group (**A**) and matched antibody concentrations per individual at T1 (blue) and T2 (red) per age in years (**B**)

**Fig. 3:**
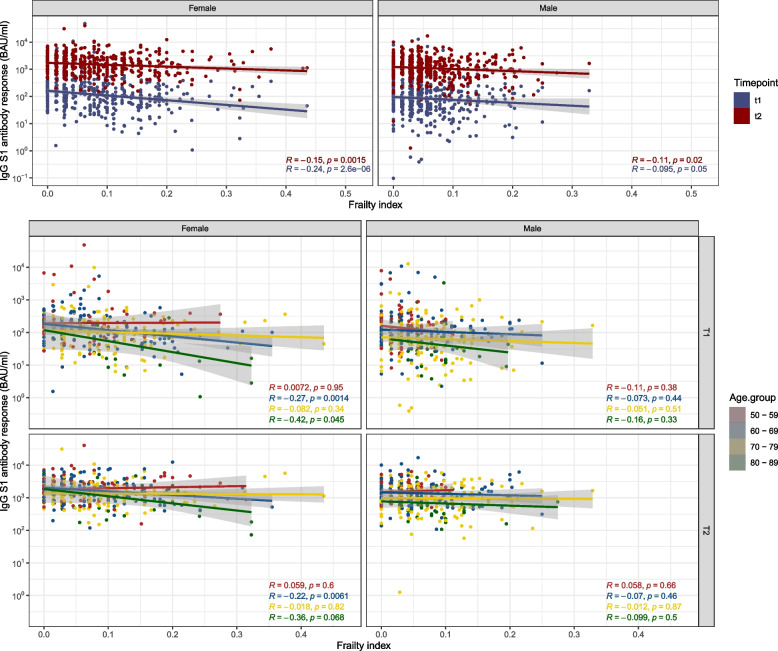
Correlations of anti-S1 IgG concentrations in binding antibody units per milliliter (BAU/ml) with a frailty index based on 36 deficits using Pearson correlation at a month post first vaccination (T1), and a month post second vaccination (T2) for men and women across all ages and per ten year age categories

At all ages anti-S1 IgG concentrations showed an increase after vaccination. However, at both timepoints higher age was associated with a lower antibody response, as can be seen in Fig. 2A (for ten-year age categories) and 2B (for age in years). The variation in the log transformed antibody response also decreased upon the second vaccination compared to the first vaccination (IQR_T1_ = 1.46, IQR _T2_ = 1.12). Although not analyzed further due to the low number of participants, similar trends can be seen for those vaccinated with AZD1222 (AstraZeneca) in Fig. S1. In Fig. 3 the Pearson correlation coefficients between the anti-S1 IgG concentrations and the frailty index can be seen. Higher levels of frailty are correlated with a lower anti-S1 antibody response at both timepoints. This means that even though the anti-S1 IgG concentration increases upon the second vaccination there was still a negative correlation between the frailty index and the IgG response for both men (*R*_T1_ = -0.095, *P*_T1_ = 0.05; *r*_T2_ = -0.11, *p*_T2_ = 0.02) and women (*R*_T1_ = -0.24, *P*_T1_ < 0.01; *R*_T2_ = -0.15, *P*_T2_ < 0.01). This can also be observed for women of age 80–89 at T1 (*R* = -0.42, *P* < 0.05) and women of age 60–69 at T1 and T2 (*R*_T1_ = -0.27, *P*_T1_ < 0.01; *R*_T2_ = -0.22, *P*_T2_ < 0.01).

### Multivariate analysis of BNT162b2 induced antibody responses corrected for age and sex

Multivariate analysis of antibody concentrations adjusted for age and sex was performed for each other variable separately while adjusting *P* values using the Benjamini-Hochberg procedure. Waist circumference (β = -0.013), BMI (β = -0.03), the frailty index (β = -2.3), HDL cholesterol concentrations (β = 0.29), kidney function based on estimated glomerular filtration rate, eGFR, (β = -0.55), lung function based on FEV1 (β = -0.17), a history of balloon dilatation (β = -0.73) or cardiac catheterization (β = -0.36), diabetes (β = -0.69), and lower back pain (β = -0.39) were all (with the exception of HDL cholesterol concentrations) negatively associated with the anti-S1 IgG concentrations at T1 (Table S3).

Looking at changes in concentrations of anti-S1 IgG, at T2, 1 month post second vaccination a history of myocardial infarction (β = 0.72), lower-back pain (β = -0.23), difficulties with household activities (β = -0.29), and a cognitive flexibility score in the lowest decile of the entire study population distribution, also further referred to as an impaired cognitive flexibility (β = -0.47), were associated with the anti-S1 IgG concentrations. Of these associations only a history of myocardial infarction showed a positive relationship with the antibody responses (Table S3).

In contrast, when looking at log-fold changes in anti-S1 IgG concentrations between T1 and T2, these variables showed associations in the opposite direction for the log-fold change between the two timepoints compared to their associations with the anti-S1 IgG response at T1 or T2. Waist circumference (β = 0.013), HDL cholesterol (β = -0.22), blood glucose concentrations (β = 0.046), the frailty index (β = 1.6), eGFR (β = 0.58), diabetes (β = 0.73), and BMI (β = 0.034) all showed associations with the log-fold change in antibody response that are in the opposite direction compared to their associations with that at a month after the vaccinations.

### Multivariate regression of BNT162b2 induced antibody responses

**Fig. 4:**
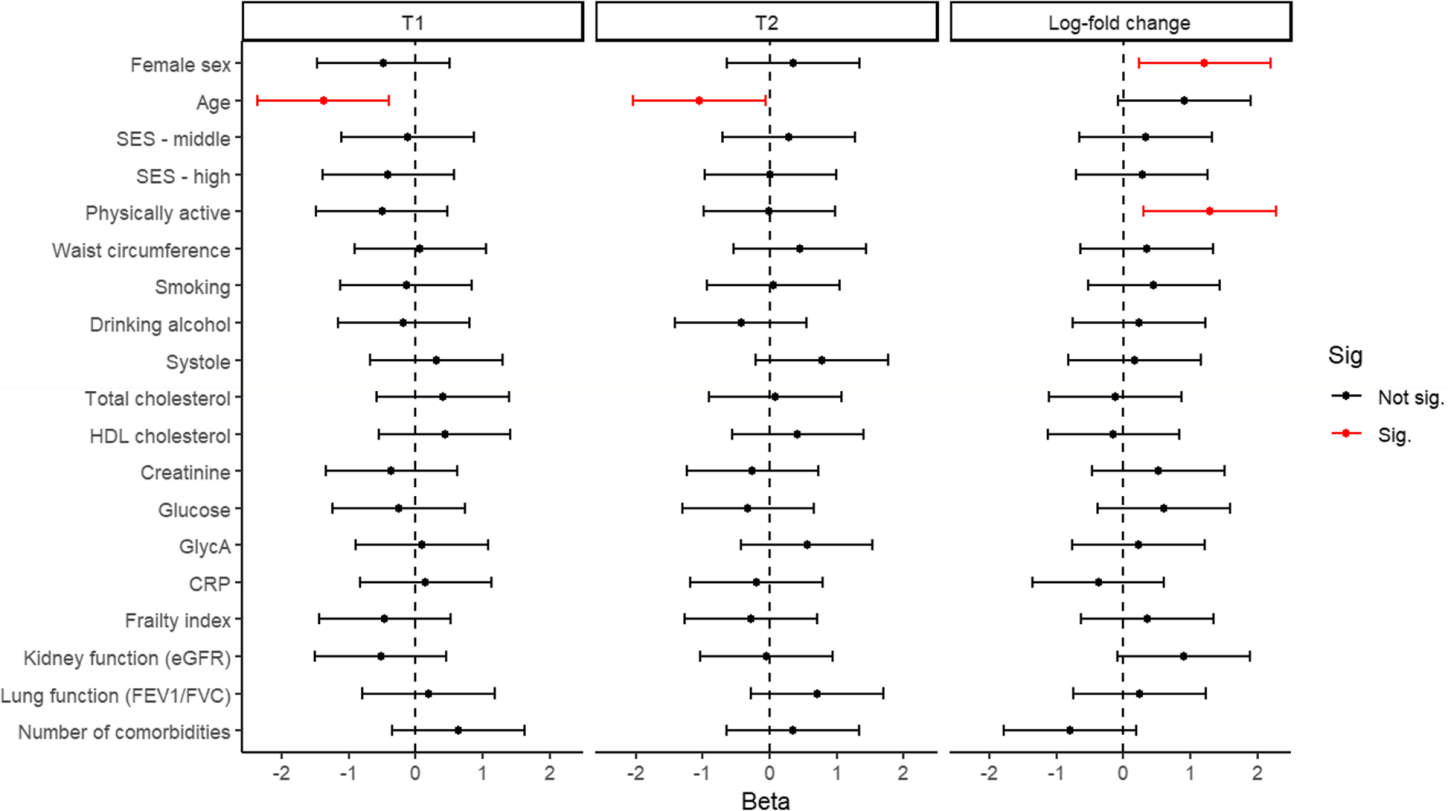
Multivariate associations and their respective 95% CI’s in case of statistical significance of sociodemographic and cardiometabolic variables with the anti-S1 IgG concentrations at T1, T2, and the log fold change between these two timepoints after mRNA BNT162b2 vaccination. Estimates have been divided by 2*SD to rescale them. Significant associations are colored red

Using multivariate modelling, sex, age, and being physically active all correlated with the anti-S1 IgG concentrations upon both vaccinations, and/or the log-fold change in IgG concentration between the two timepoints. At both T1 and T2, age correlated negatively with the anti-S1 IgG concentrations as shown in Fig. 4 and Table S4 (β_T1_ = -0.062 [-0.11, -0.018], β_T2_ = -0.031 [-0.06, -0.0023]). Both female sex and being physically active correlated positively with the log fold change in anti-S1 IgG concentrations between T1 and T2 as can be seen in Fig. 4 and Table S4 (β_female_ = 0.6 [0.05, 1.2]; β_active_ = 0.44 [0.0944, 0.79]).

**Fig. 5:**
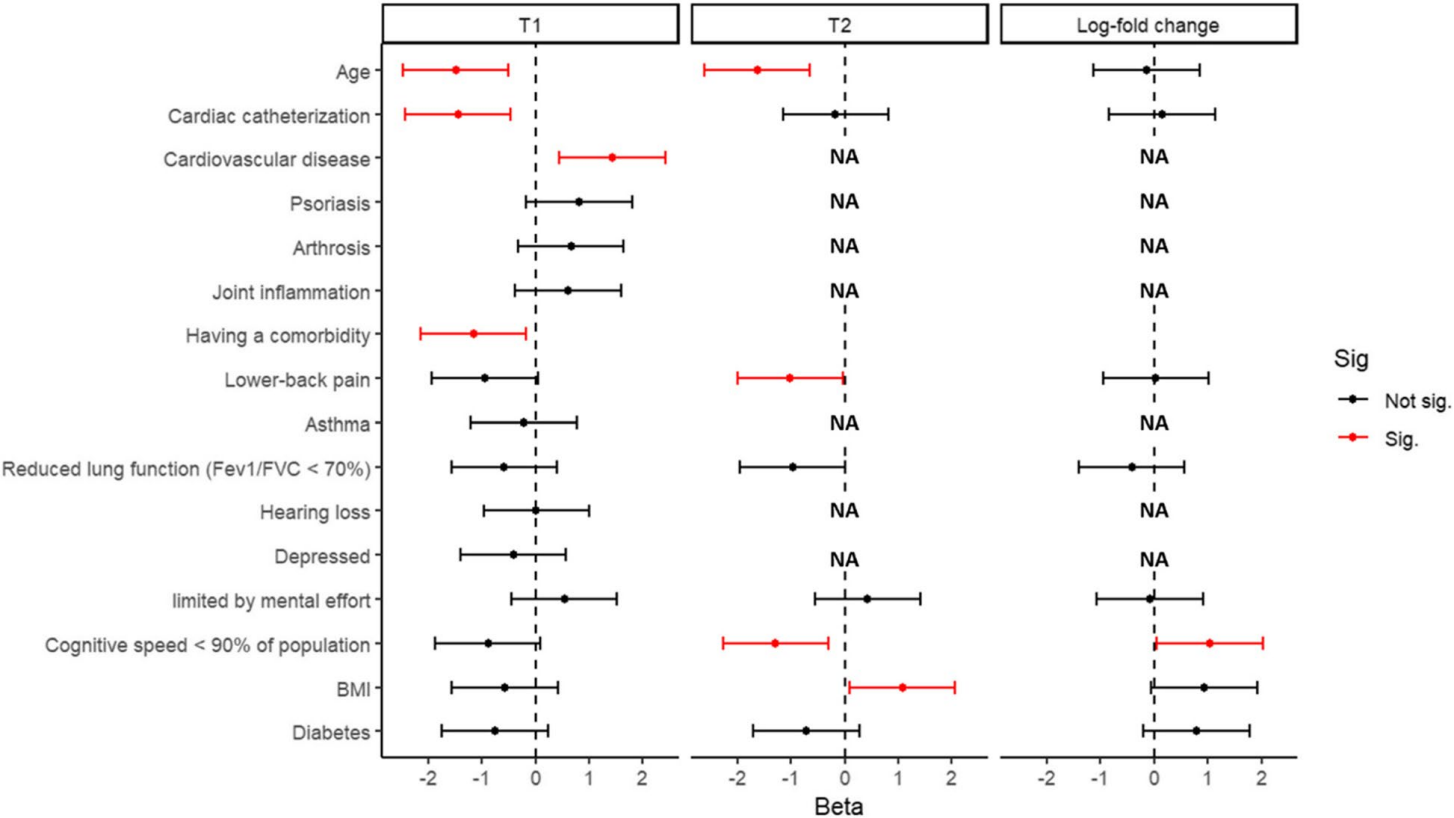
Multivariate associations and their respective 95% CI’s in case of statistical significance of comorbidities and frailty index parameters with the anti-S1 IgG concentration at T1, T2, and the log fold change between these two timepoints. Estimates have been divided by 2*SD to rescale them. Significant associations are colored red. If variables were not included in the stepwise regression model for T2 or the log-fold change “NA’s” are plotted instead

Additionally, after stepwise regression analysis using additional comorbidities and all individual components of the frailty index, several statistically significant associations with the antibody response were observed as shown in Fig. 5 and Table S5. At T1, age (β = -0.045), a history of cardiac catheterization (β = -1.9) and suffering from any comorbidity (β = -0.66) were associated with a lower anti-S1 IgG concentration. On the other hand, having hypertension or a history of various procedures for cardiovascular diseases (β = 1.7) were associated with a higher anti-S1 IgG concentration as shown in Fig. 5. At T2, age (β = -0.03), having an impaired cognitive speed (β = -0.59), a gastrointestinal disease (β = -0.65), and lower-back pain (β = -0.41), were all associated with lower anti-S1 IgG concentrations, whereas only BMI (β = 0.31), and osteoporosis (β = 0.52) were associated with higher anti-S1 IgG concentrations, as shown in Fig. 5. As for the log fold change between these two timepoints we only observed a statistically significant positive association between the relative increase in anti-S1 IgG concentrations and having an impaired cognitive speed, as can be seen in Fig. 5.

## Discussion

In this study we analyzed antibody responses to anti-SARS-CoV-2 vaccination in older persons, aiming to identify potential determinants of heterogeneity in vaccine responsiveness. While frailty as determined by a composite of multiple comorbidities and other health related variables seemingly had an important role in the primary vaccination response to the BNT162b2 vaccine within this ageing population the effect partially disappeared after correcting for age and sex. The reduced antibody response at T1 and the increase between the two vaccine doses were, however, significantly associated with the frailty index.

After adjustment for age and sex, factors that were associated with the antibody responses were physical activity, waist circumference, BMI, HDL and glucose concentrations, kidney and lung function, diabetes, a history of cardiovascular procedures, lower cognitive abilities, and more physical impairments. We observed that persons with a lower antibody response after their first vaccination also tended to have lower antibody responses after their second vaccination. These persons however had a higher relative increase in antibody concentrations upon their second vaccination, resulting in a smaller IQR of the antibody concentrations at T2 compared to T1.

Multivariate analysis showed several aspects of frailty to play a role in explaining the heterogeneity in antibody responses after the primary vaccination series against a novel pathogen. Age, BMI, a history of cardiovascular procedures, gastrointestinal disease, a reduced lung function, and impaired cognitive speed were all statistically significantly associated with the antibody concentrations.

The associations of these factors separately have been shown in other longitudinal cohort studies. Specifically, that older age, male sex, diabetes, hypertension, and heart disease are associated with a lower antibody response 1 month after vaccination with BNTT162b. This effect was reduced 1 month after the second vaccination [20, 21].

Although these studies have provided evidence that hypertension, amongst other cardiovascular diseases, is a risk factor for a reduced antibody response after vaccination, treatment against hypertension has, in contrast, been linked to clinical benefits in COVID-19. These benefits were supposedly related to improved antibody production through positive effects on inflammatory pathways and antigen presentation [22, 23]. Given the fact that the older persons in this study were treated against hypertension respectively for other cardiovascular diseases, the positive relation we see between hypertension, or cardiovascular disease, and the antibody response, could in fact be a treatment effect.

The role of physical activity in relation to immunosenescence has been studied by several researchers. In particular, remaining physically active is thought to positively affect immune function and reduce age-related comorbidities [24]. This was consistent with our findings that showed that being physically active was positively associated with the log-fold change in anti-S1 IgG response during the primary vaccination series with BNT162b.

The findings that greater cognitive speed was positively associated with anti-S1 IgG has, to our knowledge, not been reported in other studies. Studies on cognitive impairment and COVID-19 have mostly focused on either cognitive impairment as rare symptom of long-COVID or as rare side-effect of vaccination against COVID-19, i.e. not as associate of immune responses. Other studies that did focus on pre-pandemic cognitive impairment in relation to vaccines, have done so in the context of vaccination willingness and not IgG response upon vaccination [25].

A strength of our study was that we could employ an extensive data set on the study participants that had been collected prior to the vaccination, and that we could relate all these data to the antibody responses at regular moments after vaccination in a large and aging cohort. Furthermore, the fact that this data had been collected for all DCS participants, allowed us to make use of a frailty index that had been validated in this greater cohort.

Using an already existing cohort allowed us to create an extensive dataset with information regarding comorbidities, lifestyle, sociodemographic factors, and other measures of frailty and chronic inflammation. To determine the presence of certain diseases we used all available longitudinal data. This made it possible to identify potential determinants of anti-S1 IgG responses, which would have been less feasible with a newly formed cohort. Furthermore, it allowed us to evaluate markers such as the frailty index and study if these markers are indicative of the vaccine induced anti-S1 IgG response.

However, there are also limitations. Not all participants responded in time to be included at T0 or T1 and as such we had fewer samples for T1, which is where we expected to observe a larger heterogeneity in antibody responses. Additionally, we aimed to use the most recent data before vaccination for each participant separately, meaning some participants had data for as recent as early 2021 whereas for other participants data were only available for not more recent than 2013. The fact that we did not have the most recent data for all participants might have introduced some bias. Since we were looking at mostly chronic illnesses and conditions, we could expect that some individuals developed a comorbidity between their data collection and antibody measurement. Such individuals would have been misclassified and be more frail than acknowledged. Misclassification in the opposite direction is unlikely: a comorbidity reported at an older date would still be present when they had their antibody measurements taken. Ideally cohort data would have collected in such a manner that for all participants the most recent data just before the start of the pandemic would be available, but this was not feasible given the size and the logistics of the Doetinchem Cohort Study. Lastly, we also studied a relatively healthy population that was still able to participate in a study such as this one, meaning we did not capture the frailest individuals.

## Conclusions

Various factors such as age, sex, specific components of frailty, and comorbidities were associated with the anti-S1 IgG antibody response after vaccination with BNT162b2 in our ageing population. This implied a reduced antibody concentration in older and frailer persons, more specifically those with chronic comorbidities, lower cognitive speed, and greater physical impairments. Men as well as those who are physically inactive also showed a reduced increase in anti-S1 IgG response during their primary vaccination series. An increased antibody response can be seen in those who have experienced cardiac events in the past.

Among the older persons, those who were frailer and less healthy had a lower antibody response after their first vaccination yet experienced a stronger increase in their antibody response after their second vaccination compared to less frail and healthier persons. However, they still had a lower antibody response after their second vaccination compared to these less frail and healthier persons. This reduced response after completing their primary vaccination series was however no longer significant after adjusting for age and sex whereas the reduced response after one dose and the increase between the two timepoints remained significant.

This highlights the importance of studying the heterogeneous vaccine induced antibody responses in older individuals within the general population. This allows for the identification of potential risk groups with a weaker response to the vaccinations, and potentially adjusting their vaccination regimen. Furthermore, it enables the identification of factors such as being physically active which those with lower antibody responses can still affect in order to to positively impact their antibody response. Further studies are needed to assess the immunological mechanisms behind these potential risk factors affecting the vaccine responsiveness in older persons.

## Methods

### Cohort selection

We used the Doetinchem Cohort Study [18, 19], that started in 1987 with a population-based sample of men and women aged 20–59 years old who have been followed up every 5 years. The study collects data on lifestyle factors, biological measurements, physical and cognitive functioning, social aspects, comorbidities, and other background characteristics. From this cohort we invited all 3647 remaining participants to take part in the COVID-19 vaccination study. Participants were included in the study if they planned to receive COVID-19 vaccination or had completed the primary vaccination series within the last 28 days, as a month post second vaccination was the primary endpoint of the study.

The numbers of participants in the study are depicted in Fig. 1. In total 1457 DCS subjects were included in the vaccination study. As the study commenced after the start of the national vaccination campaign and vaccines were rolled-out per age group from old to young according to the national guidelines, some persons missed the pre-vaccination (T0) or even the T1 sampling. Thus, the number of individuals included in the study increased at subsequent timepoints. The median interval between the two vaccination doses was 35 days (interquartile range, IQR: 35–35) and did not differ between ages. At pre-vaccination (T0), 916 of the participants had a baseline antibody measurement taken, had complete cohort data, and were negative for COVID-19 infection. At 1 month after the first vaccination (T1) this applied to 1118 individuals and at a month after the second vaccination (T2) to 1257 individuals. Prior to receiving a vaccination 8.3% tested positive for COVID-19 and 1 month after completing the primary vaccination series this was 8.2%.

For further analysis, persons who had not yet been infected prior to vaccination or during our study (infection naive) were selected. One thousand twenty individuals were sampled at both T1 and T2. In these individuals the fold increase in antibody concentration between the two vaccinations was determined. Since the majority (78% at T1 and T2) of the participants was vaccinated with BNT162b2, the main analyses were done on this group. Persons of 60–65 years of age have mainly been vaccinated with AZD1222 (20% at T1 and T2). Therefore, the antibody response across the different timepoints has been evaluated in this subset of individuals.

#### Sample collection

Blood samples and questionnaires were taken prior to COVID-19 vaccination (T0 +7), 28 (-8 + 15) days after the first vaccination (T1), and 28 (-15 + 24 days) after the second vaccination (T2). The median interval between the two vaccination doses was 35 days (interquartile range, IQR: 35–35). Questionnaires covered demographic factors, COVID-19 vaccination information (type and date of vaccination), and SARS-CoV-2 testing information. Finger-prick blood samples were self-collected in microtubes and returned by mail. Serum was isolated from each sample by centrifugation and stored at -20°C until sample processing.

### SARS-CoV-2 IgG antibody response measurement

Immunoglobulin G (IgG) antibody concentrations against Spike S1 and Nucleoprotein (N) were measured simultaneously using a bead-based assay as previously described [26]. IgG concentrations were calibrated against the International Standard for human anti-SARS-CoV-2 immunoglobulin (20/136 NIBSC standard) and expressed as binding antibody units per milliliter (BAU/ml) [27]. The threshold for seropositivity was set at 10.1 BAU/ml for Spike S1 [28] and 14.3 BAU/ml for Nucleoprotein [29].

### Measurement of variables

Participants had filled in questionnaires relating to quality of life and general health during each 5-year follow up phase of the DCS (Round 1 – 7) prior to the vaccination study. Further data was collected covering various topics such as demographic and lifestyle factors, and comorbidities, both self-reported and confirmed by physicians. Questionnaires were sent out via mail but participants could rely on assistance from professional healthcare workers in case they requested it. Questionnaires sent out prior to the vaccination study were also validated by a professional healthcare worker and the participant during the physical examination performed in each round of the Doetinchem Cohort Study. In addition, the physical examination included measurement of blood pressure, lung function, a cognitive test battery, physical functioning, as well as taking a blood sample for measurement of total- and HDL-cholesterol, and glucose. For CRP and glycA which had been measured in stored blood samples previously, the most recent measurements were used.

### Frailty index calculation

Using the collected data a frailty index was calculated. This frailty index is a measure consisting of 36 ‘deficits’ defined based on chronic conditions, cognitive, physical, and psychological functioning as described before [30]. The 36 deficits were selected based on previous inclusion in existing frailty indexes, a prevalence of greater than one percent in the entire DCS cohort, and if there was a known association with cognitive, physical, or psychological functioning. Health deficits were either dichotomized or trichotomized with 0 indicating total absence, 0.5 indicating partial/mild presence, and 1 indicating total presence of a given deficit. The sum of deficits was then divided by the number of deficits included resulting in an index ranging from 0 (completely non-frail) to 1 (completely frail). This measure of frailty has been linked to various inflammatory markers and clinically relevant health related outcomes before within the DCS [31].

### Statistical analysis

IgG concentrations were log-transformed prior to all analyses resulting in approximately normally distributed values. In all analyses the IgG response at T1, T2, and the relative increase between these two timepoints were analyzed separately. All statistical analyses were performed using R version 4.2.0. Statistical significance was defined using a *p*-value not greater than 0.05.

To test whether frailty and age influenced both the absolute and relative vaccine induced IgG response a Pearson correlation analysis was performed. This was done to determine which specific variables, used to construct the frailty index, to include for further analysis. Linear regression models correcting for age and sex were constructed to highlight how the different frailty-related parameters as well as other comorbidities were associated with the IgG response independent of age and sex. For the resulting *P* values of these linear models a Benjamini-Hochberg correction was performed for the adjusted *P* values.

A multivariable linear regression model was constructed including several preselected variables commonly associated with clinically relevant health outcomes. These variables included age, sex, socioeconomic status, physical activity, waist circumference, smoking behavior, alcohol consumption, systolic blood pressure, (HDL) cholesterol, creatinine, glucose, glycA, and CRP concentrations, as well as kidney function, lung function, frailty index, and the number of comorbidities.

Following this, a multivariate linear regression model was constructed using all frailty-related parameters and comorbidities. First, multiple stepwise regression was performed on a subset of the samples without missing data to select which variables would be included in the regression model. This was done to identify which combination of variables led to the most parsimonious model that best explained the vaccine induced IgG. The variables selected out of these frailty-related parameters and other comorbidities were used to create a multivariate linear model using all samples available in order to estimate the effects of each of these selected variables.

### Supplementary Information

**Additional file 1: Table S1.** Prevalence of comorbidities and frailty index parameters in the study population (*N* = 1457).

**Additional file 2: Table S2.** Prevalence and mean (SD) of sociodemographic, cardiometabolic, and comorbidity related variables in the complete Doetinchem Cohort Study and the vaccination study sub-cohort.

**Additional file 3: Figure S1.** Distribution of the SARS-CoV-2 -S1 IgG antibody concentrations in binding antibody units per milliliter (BAU/ml) in persons aged 50-70 years of age vaccinated with AZD1222 at T0, T1, and T2 per ten year age group (A) and matched antibody concentrations per individual at T1 (blue) and T2 (red) across age in years (B).

**Additional file 4: Table S3.** Linear associations with anti-S1 IgG concentrations 1 month after first vaccination dose (T1), second vaccination dose (T2), and the log-fold change during the primary vaccination series with BNT162b2, corrected for age and sex. Statistically significant (*P* <= 0.05) associations are made bold.

**Additional file 5: Table S4.** Multivariate correlation of sociodemographic and cardiometabolic variables with the anti-S1 antibody concentrations 1 month after first vaccination dose (T1), second vaccination dose (T2), and the log-fold change during the primary vaccination series with BNT162b2. Statistically significant (*P* <= 0.05) associations are made bold.

**Additional file 6: Table S5.** Multivariate correlation of comorbidities and frailty index parameters with the anti-S1 antibody concentrations 1 month after first vaccination dose (T1), second vaccination dose (T2), and the log-fold change during the primary vaccination series with BNT162b2. Statistically significant (*P* <= 0.05) associations are made bold.

## Data Availability

We welcome collaboration. For use of the available data, please contact Professor W. M. M. Verschuren or H. S. J. Picavet, PhD (doetinchemstudie@rivm.nl).
