# Supplementary material for: Potential determinants of antibody responses after vaccination against SARS-CoV-2 in older persons: the Doetinchem Cohort Study
Source: Immun Ageing. 2023 Oct 25;20:57. doi: 10.1186/s12979-023-00382-4 (PMC10599057; doi:10.1186/s12979-023-00382-4)
Supplement: Supplementary file 1 — Additional file 1: Table S1. Prevalence of comorbidities and frailty index parameters in the study population (N = 1457). [file 12979_2023_382_MOESM1_ESM.docx]

**Table S1:** *Prevalence of comorbidities and frailty index parameters in the study population (N = 1457).*

| **Frailty index parameters** |  |
| --- | --- |
| *Physical* |  |
| Physically inactive* | 3.0 |
| Weak hand grip strength** (%) | 2.1 |
| Ankle brachial index (ABI <= 0.9) (%) | 0.9 |
| Underweight (BMI <=18.5 kg/m2) or obese (BMI >= 30 kg/m2)(%) | 17.0 |
| Incontinence (%) | 12.4 |
| Falling due to vertigo (%) |  |
| None | 98.2 |
| Self-reported | 1.3 |
| Confirmed by physician | 0.5 |
| Interference with normal living due to pain during past 4 weeks (%) |  |
| Not at all | 60.2 |
| A little | 37.4 |
| A lot | 2.4 |
| Limited in activities of daily living (ADL) due to poor health (%) |  |
| Not at all | 96.6 |
| A little | 2.6 |
| A lot | 0.8 |
| Limited in moderate activities due to poor health (%) |  |
| Not at all | 84.0 |
| A little | 13.5 |
| A lot | 2.5 |
| Limited while walking due to poor health (%) |  |
| Not at all | 94.9 |
| A little | 3.8 |
| A lot | 1.3 |
| Limited in vigorous activities due to poor health (%) |  |
| Not at all | 73.9 |
| A little | 22.6 |
| A lot | 3.4 |
| Limited while climbing stairs due to poor health (%) |  |
| Not at all | 91.5 |
| A little | 7.0 |
| A lot | 1.5 |
| *Cognitive factors* |  |
| Speed ranked in lowest decile in DCS cohort | 5.2 |
| Memory ranked in lowest decile in DCS cohort | 5.3 |
| Flexibility ranked in lowest decile in DCS cohort | 4.1 |
| *Psychological* |  |
| Poor self-perceived health (%) | 10.6 |
| Feeling downhearted and blue (%) |  |
| Rarely | 84.2 |
| Occasionally | 13.9 |
| Continuously | 1.9 |
| Feeling happiness (%) |  |
| Rarely | 2.9 |
| Occasionally | 23.2 |
| Continuously | 73.9 |
| Feeling a lack of energy (%) |  |
| Rarely | 73.5 |
| Occasionally | 24.6 |
| Continuously | 1.8 |
| Feeling worn out (%) |  |
| Rarely | 69.3 |
| Occasionally | 29.2 |
| Continuously | 1.4 |
| *Comorbidity related frailty parameters* |  |
| Cardiovascular disease (%) | 3.9 |
| High systolic blood pressure (>= 160 mmHg) (%) | 5.8 |
| Diabetes (%) | 6.9 |
| Self-reported malignancy (%) | 11.3 |
| Joint inflammation (%) | 10.0 |
| Osteoporosis (%) | 7.3 |
| Cerebrovascular accident (CVA) (%) | 1.2 |
| Migraine (%) | 12.7 |
| Nervous system diseases (%) | 0.8 |
| Low spirometry ratio (FEV/FVC <= 0.7) (%) | 20.7 |
| Impaired renal function (eGFR < 60) (%) | 7.8 |
| Trouble with eyesight (%) | 5.6 |
| Trouble hearing (%) | 4.5 |
| Severe lower back pain (%) |  |
| None | 84.9 |
| Self-reported | 4.9 |
| Confirmed by physician | 10.2 |
| Severe digestive tract disorders (%) |  |
| None | 95.0 |
| Self-reported | 1.4 |
| Confirmed by physician | 3.6 |
| Asthma (%) |  |
| No Asthma | 94.0 |
| Asthma without attacks | 5.3 |
| Asthma with attacks | 0.7 |
| **Other comorbidities** |  |
| Arthrosis (%) | 29.6 |
| Psoriasis (%) | 6.4 |
| Ever high blood pressure (%) | 48.2 |
| Hypertension (%) | 47.4 |
| Myocardial infarction (%) | 3.3 |
| Coronary artery bypass (%) | 2.1 |
| Balloon dilation (%) | 2.6 |
| Cardiac catheterization (%) | 7.0 |
| Pacemaker (%) | 1.4 |
| Vascular surgery (%) | 1.4 |
| Any comorbidity (%) | 54.9 |

* Physically inactive is based on failing to meet the Dutch healthy exercise norm, belonging to the 25^th^ lowest percentile of walking activity, and belonging to the 10^th^ percentile lowest low/medium/high intensive activities in the DCS.

** Hand grip strength is adjusted for sex and BMI, with weak hand grip strength referring to a handgrip strength of <17 through >21 kg for women with a BMI ranging between <23 through >29. And a hand grip strength of <29 through >32 kg for men with a BMI ranging between <24 through >28.
