# Supplementary material for: Potential determinants of antibody responses after vaccination against SARS-CoV-2 in older persons: the Doetinchem Cohort Study
Source: Immun Ageing. 2023 Oct 25;20:57. doi: 10.1186/s12979-023-00382-4 (PMC10599057; doi:10.1186/s12979-023-00382-4)
Supplement: Supplementary file 2 — Additional file 2: Table S2. Prevalence and mean (SD) of sociodemographic, cardiometabolic, and comorbidity related variables in the complete Doetinchem Cohort Study and the vaccination study sub-cohort. [file 12979_2023_382_MOESM2_ESM.docx]

**Table S2:** Prevalence and mean (SD) of sociodemographic, cardiometabolic, and comorbidity related variables in the complete Doetinchem Cohort Study and the vaccination study sub-cohort.

|  | N=3647 | N=1457 |
| --- | --- | --- |
| **Sociodemographic** |  |  |
| Women (%) | 53 | 51.2 |
| Age (years) (mean (SD)) | 65.8 (9.4) | 67.4 (7.7) |
| Socio-Economic Status (%) |  |  |
| *Low* | 44 | 33 |
| *Middle* | 30 | 35 |
| *High* | 26 | 32 |
| ***Lifestyle*** |  |  |
| Current smokers (%) | 7.4 | 6.6 |
| Drinking alcohol (%) | 61 | 68 |
| Adherence to Dutch healthy exercise norm (NNGB) (%) | 60 | 63 |
| **Cardiometabolic factors** |  |  |
| Waist circumference (cm) (mean (SD)) | 97.0 (12.5) | 96.4 (12.3) |
| BMI (kg/m^2^) (mean (SD)) | 26.8 (4.3) | 26.6 (4.2) |
| Systolic blood pressure (mmHg) (mean (SD)) | 132 (17) | 131 (17) |
| Total cholesterol (mmol/L) (mean (SD)) | 5.4 (1.1) | 5.4 (1.0) |
| HDL cholesterol (mmol/L) (mean (SD)) | 1.5 (0.4) | 1.5 (0.4) |
| Creatinine (mmol/L) (mean (SD)) | 82.5 (20.0) | 82.3 (19.0) |
| Glucose (mmol/L) (mean (SD)) | 5.9 (1.7) | 5.7 (1.6) |
| GlycA (mmol/L) (mean (SD)) | 0.9 (0.1) | 0.9 (0.1) |
| CRP (mmol/L) (mean (SD)) | 2.3 (4.0) | 2.2 (4.6) |
| **Comorbidity related variables** |  |  |
| Frailty index (median (IQR)) | 0.10 (0.03 – 0.14) | 0.06 (0.03 – 0.11) |
| FEV1 max (mean (SD)) | 3.0 (0.8) | 3.1 (0.8) |
| FVC max (mean (SD)) | 4.0 (1.1) | 4.2 (1.0) |
| FEV1/FVC ratio (mean (SD)) | 0.7 (0.1) | 0.7 (0.1) |
| eGFR (mean (SD)) | 0.9 (0.3) | 0.9 (0.3) |
| Having >=1 comorbidity (%) | 63 | 55 |
