# Supplementary material for: Potential determinants of antibody responses after vaccination against SARS-CoV-2 in older persons: the Doetinchem Cohort Study
Source: Immun Ageing. 2023 Oct 25;20:57. doi: 10.1186/s12979-023-00382-4 (PMC10599057; doi:10.1186/s12979-023-00382-4)
Supplement: Supplementary file 3 — Additional file 3: Figure S1. Distribution of the SARS-CoV-2 -S1 IgG antibody concentrations in binding antibody units per milliliter (BAU/ml) in persons aged 50-70 years of age vaccinated with AZD1222 at T0, T1, and T2 per ten year age group (A) and matched antibody concentrations per individual at T1 (blue) and T2 (red) across age in years (B). [file 12979_2023_382_MOESM3_ESM.docx]

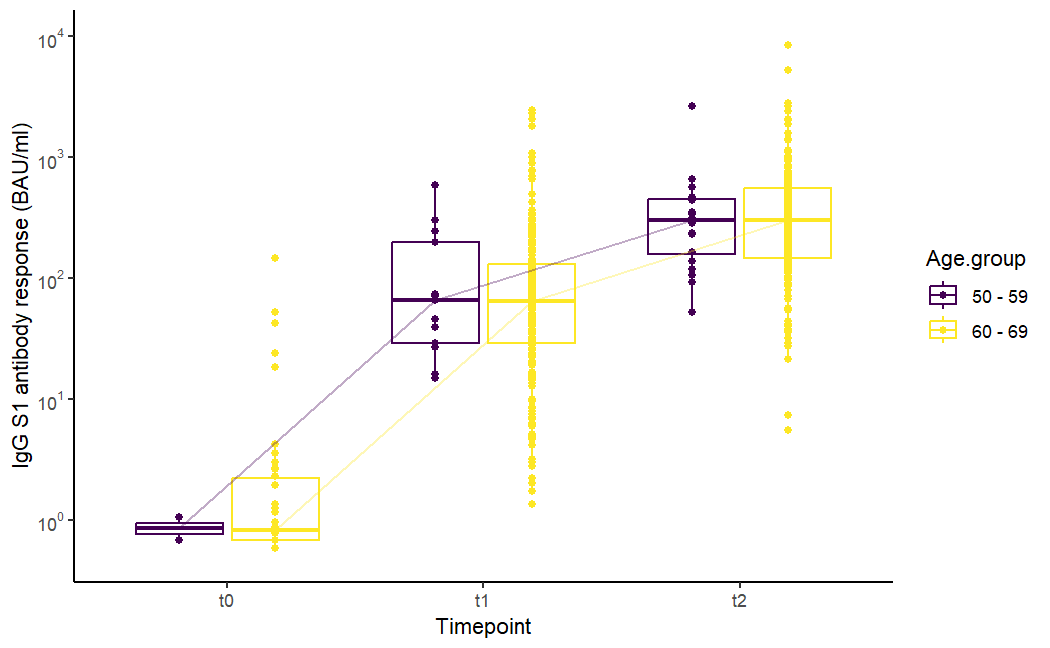

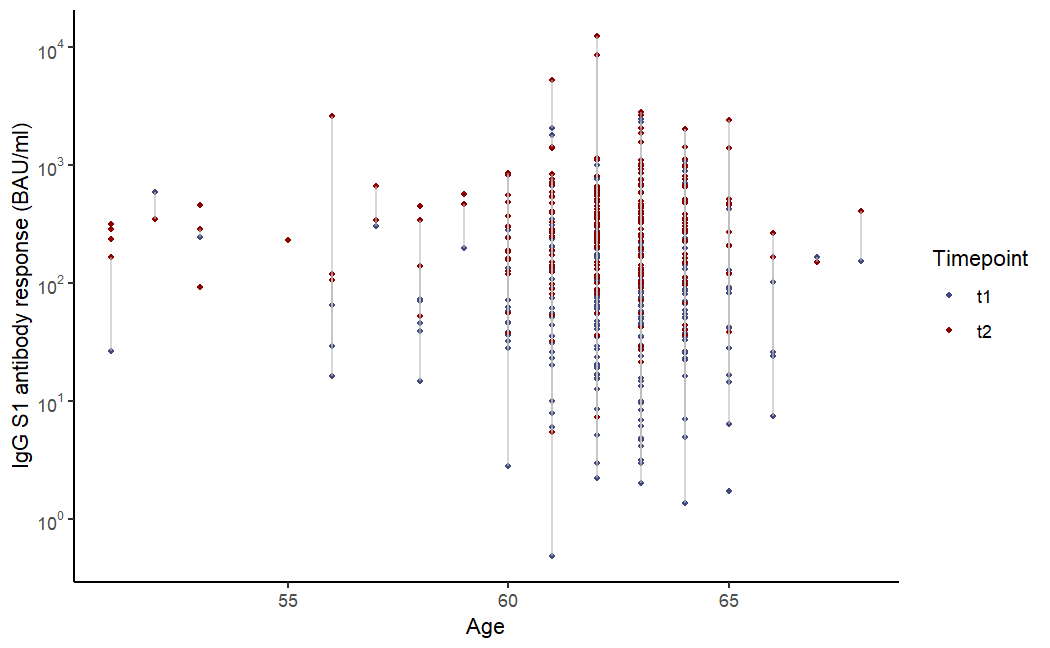


A

B

**Figure S1:** *Distribution of the SARS-CoV-2 -S1 IgG antibody concentrations in binding antibody units per milliliter (BAU/ml) in persons aged 50-70 years of age vaccinated with AZD1222 at T0, T1, and T2 per ten year age group (A) and matched antibody concentrations per individual at T1 (blue) and T2 (red) across age in years (B).*
