# Supplementary material for: Potential determinants of antibody responses after vaccination against SARS-CoV-2 in older persons: the Doetinchem Cohort Study
Source: Immun Ageing. 2023 Oct 25;20:57. doi: 10.1186/s12979-023-00382-4 (PMC10599057; doi:10.1186/s12979-023-00382-4)
Supplement: Supplementary file 4 — Additional file 4: Table S3. Linear associations with anti-S1 IgG concentrations 1 month after first vaccination dose (T1), second vaccination dose (T2), and the log-fold change during the primary vaccination series with BNT162b2, corrected for age and sex. Statistically significant (P <= 0.05) associations are made bold. [file 12979_2023_382_MOESM4_ESM.docx]

**Table S3:** *Linear associations with anti-S1 IgG concentrations one month after first vaccination dose (T1), second vaccination dose (T2), and the log-fold change during the primary vaccination series with BNT162b2, corrected for age and sex. Statistically significant (P <= 0.05) associations are made bold.*

|  | ***T1: N=853*** | | | | ***T2: N=954*** | | | | ***Log-fold change: N=791*** | | | |
| --- | --- | --- | --- | --- | --- | --- | --- | --- | --- | --- | --- | --- |
|  | **β** | **95% CI** | **P** | **P adj.** | **β** | **95% CI** | **P** | **P adj.** | **β** | **95% CI** | **P** | **P adj.** |
| *Middle socioeconomic status* | *-6.46E-02* | *-0.2910, 0.1618* | *0.58* | 0.9 | *-3.16E-02* | *-0.1755, 0.1122* | *0.67* | 0.9 | *1.49E-02* | *-0.1639, 0.1938* | *0.87* | 0.99 |
| *High socioeconomic status* | *-7.95E-02* | *-0.3014, 0.1424* | *0.48* | 0.9 | *-6.92E-02* | *-0.2125, 0.07416* | *0.34* | 0.89 | *5.34E-04* | *-0.1747, 0.1757* | *1* | 1 |
| *Physically active (NNGB)* | *-4.80E-02* | *-0.23616, 0.14016* | *0.62* | 0.9 | *1.40E-02* | *-0.10556, 0.13356* | *0.83* | 0.93 | *1.30E-01* | *-0.01896, 0.27896* | *0.089* | 0.67 |
| *Waist circumference* | ***-1.30E-02*** | ***-0.021036, -0.004964*** | ***0.0011*** | **0.046** | *5.40E-04* | *-0.004556, 0.005636* | *0.84* | 0.93 | ***1.30E-02*** | ***0.006728, 0.019272*** | ***0.00004*** | **0.0027** |
| *Currently smoking* | *-1.90E-01* | *-0.9152, 0.5352* | *0.61* | 0.9 | *-1.20E-01* | *-0.5904, 0.3504* | *0.63* | 0.9 | *1.20E-01* | *-0.468, 0.708* | *0.7* | 0.91 |
| *Drinking alcohol* | *-3.90E-02* | *-0.235, 0.157* | *0.7* | 0,90 | *-9.70E-02* | *-0.2244, 0.0304* | *0.14* | 0.74 | *-1.50E-03* | *-0.16026, 0.15726* | *0.99* | 1 |
| *Systolic blood pressure* | *2.40E-03* | *-0.003284, 0.008084* | *0.4* | 0.87 | *1.60E-03* | *-0.002124, 0.005324* | *0.4* | 0.89 | *-1.50E-03* | *-0.006008, 0.003008* | *0.52* | 0.83 |
| *Total cholesterol* | *6.40E-02* | *-0.0242, 0.1522* | *0.16* | 0.71 | *1.80E-02* | *-0.03884, 0.07484* | *0.54* | 0.9 | *-2.20E-02* | *-0.09256, 0.04856* | *0.54* | 0.83 |
| *HDL cholesterol* | ***2.90E-01*** | ***0.0548, 0.5252*** | ***0.016*** | **0.19** | *6.50E-02* | *-0.08788, 0.21788* | *0.4* | 0.89 | ***-2.20E-01*** | ***-0.4062, -0.0338*** | ***0.02*** | **0.22** |
| *Creatinine* | *-4.20E-03* | *-0.010668, 0.002268* | *0.21* | 0.83 | *-3.30E-03* | *-0.007612, 0.001012* | *0.13* | 0.74 | *2.00E-03* | *-0.003096, 0.007096* | *0.44* | 0.83 |
| *Glucose* | *-5.70E-02* | *-0.11384, -0.00016* | *0.051* | 0.29 | *-2.60E-02* | *-0.0652, 0.0132* | *0.19* | 0.74 | ***4.60E-02*** | ***0.00092, 0.09108*** | ***0.048*** | **0.47** |
| *GlycA* | *-3.70E-01* | *-1.1736, 0.4336* | *0.37* | 0.87 | *2.00E-01* | *-0.3096, 0.7096* | *0.44* | 0.89 | *3.30E-01* | *-0.2972, 0.9572* | *0.3* | 0.83 |
| *CRP* | *7.50E-03* | *-0.01406, 0.02906* | *0.48* | 0.9 | *3.70E-03* | *-0.009824, 0.017224* | *0.59* | 0.9 | *-4.80E-03* | *-0.020872, 0.011272* | *0.56* | 0.83 |
| *Frailty index* | ***-2.30E+00*** | ***-3.7112, -0.8888*** | ***0.0014*** | **0.046** | *-8.10E-01* | *-1.7116, 0.0916* | *0.081* | 0.74 | ***1.60E+00*** | ***0.4828, 2.7172*** | ***0.0051*** | **0.086** |
| *Kidney function (eGFR)* | ***-5.50E-01*** | ***-1.0204, -0.0796*** | ***0.021*** | **0.19** | *8.70E-02* | *-0.207, 0.381* | *0.57* | 0.9 | ***5.80E-01*** | ***0.2076, 0.9524*** | ***0.0024*** | **0.054** |
| *FEV1 max* | ***-1.70E-01*** | ***-0.3366, -0.0034*** | ***0.047*** | **0.29** | *-7.40E-02* | *-0.18376, 0.03576* | *0.19* | 0.74 | *6.50E-02* | *-0.06828, 0.19828* | *0.34* | 0.83 |
| *FVC max* | *-6.50E-02* | *-0.19632, 0.06632* | *0.33* | 0.87 | *-5.10E-02* | *-0.13528, 0.03328* | *0.24* | 0.81 | *-5.50E-03* | *-0.10938, 0.09838* | *0.92* | 0.99 |
| *Lung function (FEV1/FVC)* | *-6.40E-01* | *-1.9336, 0.6536* | *0.33* | 0.87 | *3.40E-01* | *-0.5028, 1.1828* | *0.43* | 0.89 | *9.30E-01* | *-0.1284, 1.9884* | *0.083* | 0.67 |
| *Number of comorbidities* | *-1.20E-02* | *-0.07668, 0.05268* | *0.73* | 0.93 | *1.80E-02* | *-0.02512, 0.06112* | *0.41* | 0.89 | *1.40E-02* | *-0.03892, 0.06692* | *0.59* | 0.91 |
| *Having any comorbidity* | *-7.70E-02* | *-0.26712, 0.11312* | *0.43* | 0.9 | *-2.20E-02* | *-0.14352, 0.09952* | *0.73* | 0.9 | *3.60E-02* | *-0.11492, 0.18692* | *0.64* | 0.97 |
| *Bypass* | *-1.30E-01* | *-0.7768, 0.5168* | *0.69* | 0.9 | *2.00E-01* | *-0.2116, 0.6116* | *0.34* | 0.89 | *1.40E-01* | *-0.35, 0.63* | *0.59* | 0.83 |
| *Balloon dilation* | ***-7.30E-01*** | ***-1.3376, -0.1224*** | ***0.02*** | **0.19** | *-3.30E-01* | *-0.7024, 0.0424* | *0.087* | 0.74 | *2.80E-01* | *-0.1904, 0.7504* | *0.25* | 0.8 |
| *Cardiac catheterization* | ***-3.60E-01*** | ***-0.7128, -0.0072*** | ***0.048*** | **0.29** | *-8.30E-02* | *-0.2986, 0.1326* | *0.47* | 0.9 | *2.40E-01* | *-0.0344, 0.5144* | *0.098* | 0.67 |
| *Pacemaker* | *-1.20E-01* | *-0.8256, 0.5856* | *0.74* | 0.91 | *-3.00E-02* | *-0.4612, 0.4012* | *0.89* | 0.95 | *1.60E-01* | *-0.3692, 0.6892* | *0.56* | 0.83 |
| *Vascular surgery* | *-1.90E-01* | *-0.974, 0.594* | *0.64* | 0.9 | *-3.30E-01* | *-0.82, 0.16* | *0.18* | 0.74 | *-2.50E-01* | *-0.8576, 0.3576* | *0.42* | 0.83 |
| *Cardiovascular disease* | *1.10E-01* | *-0.3408, 0.5608* | *0.65* | 0.9 | *7.20E-02* | *-0.222, 0.366* | *0.62* | 0.9 | *-1.40E-01* | *-0.4928, 0.2128* | *0.44* | 0.83 |
| *Myocardial infarction* | *5.60E-01* | *-0.5572, 1.6772* | *0.32* | 0.87 | ***7.20E-01*** | ***0.0732, 1.3668*** | ***0.03*** | **0.67** | *-2.90E-03* | *-0.8653, 0.8595* | *0.99* | 1 |
| *Stroke* | *2.20E-02* | *-0.8992, 0.9432* | *0.96* | 0.98 | *-9.00E-02* | *-0.7172, 0.5372* | *0.77* | 0.93 | *-7.50E-02* | *-0.8002, 0.6502* | *0.84* | 0.97 |
| *Ever high blood pressure* | *2.00E-02* | *-0.3328, 0.3728* | *0.91* | 0.97 | *1.50E-01* | *-0.0656, 0.3656* | *0.18* | 0.74 | *7.40E-02* | *-0.2004, 0.3484* | *0.61* | 0.83 |
| *Diabetes* | ***-6.90E-01*** | ***-1.3564, -0.0236*** | ***0.043*** | 0.29 | *-2.20E-01* | *-0.6708, 0.2308* | *0.33* | 0.89 | ***7.30E-01*** | ***0.1812, 1.2788*** | ***0.0094*** | **0.13** |
| *Hypertension* | *-1.20E-02* | *-0.20016, 0.17616* | *0.9* | 0.97 | *1.30E-02* | *-0.10852, 0.13452* | *0.84* | 0.93 | *2.90E-02* | *-0.11996, 0.17796* | *0.71* | 0.91 |
| *Migraine* | *-3.00E-01* | *-0.79, 0.19* | *0.24* | 0.87 | *-6.60E-02* | *-0.3992, 0.2672* | *0.7* | 0.9 | *1.90E-01* | *-0.2216, 0.6016* | *0.38* | 0.83 |
| *Gastrointestinal disease* | *-6.80E-01* | *-1.464, 0.104* | *0.092* | 0.45 | *-4.80E-01* | *-1.0484, 0.0884* | *0.1* | 0.74 | *4.60E-01* | *-0.2456, 1.1656* | *0.2* | 0.8 |
| *Psoriasis* | *2.00E-01* | *-0.4664, 0.8664* | *0.55* | 0.9 | *1.20E-01* | *-0.3308, 0.5708* | *0.59* | 0.9 | *5.10E-03* | *-0.5241, 0.5343* | *0.98* | 1 |
| *Incontinence* | *1.10E-01* | *-0.38, 0.6* | *0.67* | 0.9 | *-7.30E-02* | *-0.4062, 0.2602* | *0.66* | 0.9 | *2.50E-02* | *-0.3866, 0.4366* | *0.9* | 0.99 |
| *Hernia* | *1.10E-01* | *-0.4192, 0.6392* | *0.67* | 0.9 | *1.00E-01* | *-0.2528, 0.4528* | *0.58* | 0.9 | *-7.70E-02* | *-0.5082, 0.3542* | *0.73* | 0.91 |
| *Arthrosis* | *1.80E-01* | *-0.1924, 0.5524* | *0.36* | 0.87 | *1.60E-01* | *-0.0948, 0.4148* | *0.21* | 0.74 | *-1.00E-01* | *-0.4136, 0.2136* | *0.52* | 0.83 |
| *Joint inflammation* | *-9.10E-02* | *-0.6594, 0.4774* | *0.75* | 0.91 | *-2.00E-01* | *-0.5724, 0.1724* | *0.3* | 0.89 | *-2.80E-01* | *-0.7504, 0.1904* | *0.24* | 0.8 |
| *Osteoporosis* | *-1.50E-03* | *-0.6483, 0.6453* | *1* | 1 | *3.30E-01* | *-0.1208, 0.7808* | *0.14* | 0.74 | *-1.70E-01* | *-0.7188, 0.3788* | *0.54* | 0.83 |
| *Nervous system diseases* | *-7.70E-01* | *-2.6712, 1.1312* | *0.43* | 0.87 | *4.30E-01* | *-1.3536, 2.2136* | *0.64* | 0.89 | *8.50E-02* | *-2.071, 2.241* | *0.94* | 0.99 |
| *Falling due to vertigo* | *-2.50E-01* | *-1.1124, 0.6124* | *0.57* | 0.9 | *-4.20E-01* | *-0.9492, 0.1092* | *0.12* | 0.74 | *-2.10E-01* | *-0.8764, 0.4564* | *0.54* | 0.83 |
| *High blood pressure now* | *-1.00E-01* | *-0.4528, 0.2528* | *0.58* | 0.9 | *2.20E-02* | *-0.2132, 0.2572* | *0.85* | 0.93 | *1.40E-01* | *-0.1344, 0.4144* | *0.34* | 0.83 |
| *Malignancy* | *2.20E-02* | *-0.2524, 0.2964* | *0.88* | 0.96 | *-1.70E-02* | *-0.19732, 0.16332* | *0.86* | 0.93 | *2.50E-02* | *-0.1906, 0.2406* | *0.83* | 0.97 |
| *Lower back pain* | ***-3.90E-01*** | ***-0.684, -0.096*** | ***0.011*** | **0.18** | ***-2.30E-01*** | ***-0.42012, -0.03988*** | ***0.019*** | **0.63** | *1.80E-01* | *-0.0552, 0.4152* | *0.14* | 0.8 |
| *Cerebrovascular accident* | *-3.40E-01* | *-1.3592, 0.6792* | *0.51* | 0.9 | *2.20E-02* | *-0.664, 0.708* | *0.95* | 0.99 | *3.20E-01* | *-0.5228, 1.1628* | *0.46* | 0.83 |
| *Neurologic disease* | *-6.90E-01* | *-1.7876, 0.4076* | *0.22* | 0.83 | *-2.90E-01* | *-1.0348, 0.4548* | *0.45* | 0.89 | *4.80E-01* | *-0.4608, 1.4208* | *0.32* | 0.83 |
| *Asthma* | *2.60E-01* | *-0.4064, 0.9264* | *0.44* | 0.87 | *4.00E-01* | *-0.0312, 0.8312* | *0.071* | 0.74 | *2.20E-01* | *-0.3092, 0.7492* | *0.43* | 0.83 |
| *Impaired eyesight* | *-1.50E-01* | *-0.5224, 0.2224* | *0.43* | 0.87 | *1.00E-01* | *-0.1548, 0.3548* | *0.44* | 0.89 | *8.20E-02* | *-0.2316, 0.3956* | *0.61* | 0.83 |
| *Impaired hearing* | *1.70E-02* | *-0.4338, 0.4678* | *0.94* | 0.97 | *-5.60E-02* | *-0.3304, 0.2184* | *0.7* | 0.9 | *1.90E-02* | *-0.3534, 0.3914* | *0.92* | 1 |
| *Feeling downhearted* | *-2.90E-01* | *-0.7016, 0.1216* | *0.17* | 0.73 | *-1.80E-01* | *-0.4544, 0.0944* | *0.2* | 0.74 | *1.60E-01* | *-0.1732, 0.4932* | *0.36* | 0.83 |
| *Feeling happiness* | *-4.10E-02* | *-0.3938, 0.3118* | *0.82* | 0.93 | *1.20E-01* | *-0.1152, 0.3552* | *0.32* | 0.89 | *1.60E-01* | *-0.134, 0.454* | *0.27* | 0.8 |
| *Feeling a lack of energy* | *-1.60E-01* | *-0.5324, 0.2124* | *0.4* | 0.87 | *2.60E-02* | *-0.2092, 0.2612* | *0.84* | 0.93 | *2.30E-01* | *-0.064, 0.524* | *0.14* | 0.8 |
| *Feeling worn out* | *-2.10E-01* | *-0.5824, 0.1624* | *0.28* | 0.87 | *-1.10E-02* | *-0.2462, 0.2242* | *0.93* | 0.98 | *1.30E-01* | *-0.164, 0.424* | *0.41* | 0.83 |
| *Physically inactive* | *2.00E-01* | *-0.3488, 0.7488* | *0.48* | 0.9 | *-5.90E-03* | *-0.3587, 0.3469* | *0.97* | 0.99 | *-3.10E-01* | *-0.7412, 0.1212* | *0.16* | 0.8 |
| *Limited in ADL* | *-3.00E-01* | *-1.0448, 0.4448* | *0.43* | 0.87 | *1.10E-01* | *-0.4388, 0.6588* | *0.7* | 0.9 | *4.40E-01* | *-0.2068, 1.0868* | *0.19* | 0.8 |
| *Limited in moderate activities* | ***-5.00E-01*** | ***-0.9312, -0.0688*** | ***0.022*** | 0.19 | ***-2.90E-01*** | ***-0.5644, -0.0156*** | ***0.039*** | **0.67** | *1.90E-01* | *-0.1432, 0.5232* | *0.27* | 0.8 |
| *Limited while walking* | *-3.00E-02* | *-0.6572, 0.5972* | *0.93* | 0.97 | *-7.00E-02* | *-0.5208, 0.3808* | *0.76* | 0.93 | *2.00E-01* | *-0.3488, 0.7488* | *0.48* | 0.83 |
| *Limited in vigorous activities* | *-3.60E-01* | *-0.7324, 0.0124* | *0.06* | 0.31 | *-1.70E-01* | *-0.4052, 0.0652* | *0.18* | 0.74 | *2.00E-01* | *-0.1136, 0.5136* | *0.2* | 0.8 |
| *Limited while climbing stairs* | *-6.40E-02* | *-0.5932, 0.4652* | *0.82* | 0.93 | *1.30E-01* | *-0.2424, 0.5024* | *0.5* | 0.9 | *2.80E-01* | *-0.1708, 0.7308* | *0.22* | 0.8 |
| *Grip strength* | *1.70E-01* | *-0.4768, 0.8168* | *0.61* | 0.9 | *2.60E-03* | *-0.4286, 0.4338* | *0.99* | 0.99 | *-8.90E-02* | *-0.5986, 0.4206* | *0.73* | 0.91 |
| *Cognition: Speed <= 10th population percentile* | *-1.20E-01* | *-0.6492, 0.4092* | *0.65* | 0.9 | *-7.10E-02* | *-0.4238, 0.2818* | *0.7* | 0.9 | *2.70E-01* | *-0.1416, 0.6816* | *0.21* | 0.8 |
| *Cognition: Memory <= 10th population percentile* | *9.70E-02* | *-0.4126, 0.6066* | *0.71* | 0.9 | *-1.80E-01* | *-0.4936, 0.1336* | *0.26* | 0.85 | *-1.20E-01* | *-0.512, 0.272* | *0.57* | 0.83 |
| *Cognition: Flexibility <= 10th population percentile* | *-3.40E-01* | *-0.9476, 0.2676* | *0.27* | 0.87 | ***-4.70E-01*** | ***-0.862, -0.078*** | ***0.019*** | **0.63** | *5.00E-02* | *-0.44, 0.54* | *0.84* | 0.97 |
| *BMI* | ***-3.00E-02*** | ***-0.05156, -0.00844*** | ***0.0061*** | **0.14** | *4.20E-03* | *-0.00952, 0.01792* | *0.55* | 0.9 | ***3.40E-02*** | ***0.017144, 0.050856*** | ***0.000089*** | **0.003** |
| *Pain* | *-1.80E-01* | *-0.5132, 0.1532* | *0.31* | 0.87 | *-8.90E-02* | *-0.3046, 0.1266* | *0.43* | 0.89 | *1.20E-01* | *-0.1544, 0.3944* | *0.39* | 0.83 |
| *Ankle brachial index* | *9.90E-02* | *-1.0182, 1.2162* | *0.86* | 0.96 | *1.80E-01* | *-0.408, 0.768* | *0.54* | 0.9 | *2.40E-01* | *-0.6028, 1.0828* | *0.57* | 0.83 |
| *Poor self-perceived health* | *-1.30E-01* | *-0.4436, 0.1836* | *0.41* | 0.87 | *-2.00E-02* | *-0.216, 0.176* | *0.85* | 0.93 | *1.50E-01* | *-0.1048, 0.4048* | *0.24* | 0.8 |
