# Supplementary material for: Potential determinants of antibody responses after vaccination against SARS-CoV-2 in older persons: the Doetinchem Cohort Study
Source: Immun Ageing. 2023 Oct 25;20:57. doi: 10.1186/s12979-023-00382-4 (PMC10599057; doi:10.1186/s12979-023-00382-4)
Supplement: Supplementary file 5 — Additional file 5: Table S4. Multivariate correlation of sociodemographic and cardiometabolic variables with the anti-S1 antibody concentrations 1 month after first vaccination dose (T1), second vaccination dose (T2), and the log-fold change during the primary vaccination series with BNT162b2. Statistically significant (P <= 0.05) associations are made bold. [file 12979_2023_382_MOESM5_ESM.docx]

**Table S4:** *Multivariate correlation of sociodemographic and cardiometabolic variables with the anti-S1 antibody concentrations one month after first vaccination dose (T1), second vaccination dose (T2), and the log-fold change during the primary vaccination series with BNT162b2. Statistically significant (P <= 0.05) associations are made bold.*

|  | ***T1: N=853*** | | | ***T2: N=954*** | | | ***Log-fold change: N=791*** | | |
| --- | --- | --- | --- | --- | --- | --- | --- | --- | --- |
|  | **β** | **95% CI** | **P** | **β** | **95% CI** | **P** | **β** | **95% CI** | **P** |
| **Female sex** | -3.80E-01 | -1.1, 3.2E-01 | 0.28 | 1.10E-01 | -3.5E-01, 5.7E-01 | 0.63 | **6.00E-01** | **5.0E-02, 1.2** | **0.033** |
| **Age** | **-6.20E-02** | **-1.1E-01, -1.8E-02** | **0.0058** | **-3.10E-02** | **-6.0E-02, -2.3E-03** | **0.034** | 3.20E-02 | -3.4E-03, 6.6E-02 | 0.076 |
| **Middle socio-economic status** | -5.40E-02 | -5.5E-01, 4.4E-01 | 0.83 | 8.70E-02 | -2.2E-01, 4.0E-01 | 0.58 | 1.20E-01 | -2.7E-01, 5.0E-01 | 0.54 |
| **High socio-economic status** | -1.90E-01 | -6.8E-01, 3.0E-01 | 0.44 | -7.20E-03 | -3.2E-01, 3.1E-01 | 0.96 | 9.50E-02 | -2.9E-01, 4.8E-01 | 0.62 |
| **Physically active (NNGB)** | -2.30E-01 | -6.7E-01, 2.1E-01 | 0.3 | -1.70E-02 | -2.9E-01, 2.6E-01 | 0.91 | **4.40E-01** | **9.4E-02, 7.9E-01** | **0.013** |
| **Waist circumference (per 10cm)** | -1.80E-03 | -3.3E-01, 3.2E-01 | 0.99 | 8.10E-02 | -1.2E-01, 2.8E-01 | 0.43 | 8.40E-02 | -1.6E-01, 3.3E-01 | 0.51 |
| **Current smoker** | -1.50E-01 | -1.0, 7.3E-01 | 0.74 | -3.00E-03 | -5.4E-01, 5.3E-01 | 0.99 | 2.80E-01 | -3.7E-01, 9.4E-01 | 0.4 |
| **Alcohol consumption** | -8.00E-02 | -5.2E-01, 3.6E-01 | 0.72 | -1.10E-01 | -3.9E-01, 1.6E-01 | 0.42 | 8.10E-02 | -2.7E-01, 4.3E-01 | 0.65 |
| **Systole (per 10 mmHg)** | 3.80E-02 | -8.8E-02, 1.6E-01 | 0.55 | 6.30E-02 | -1.7E-02, 1.4E-01 | 0.12 | 1.60E-02 | -8.1E-02, 1.1E-01 | 0.75 |
| **Cholesterol (mmol/L)** | 8.70E-02 | -1.2E-01, 2.9E-01 | 0.41 | 1.50E-02 | -1.1E-01, 1.4E-01 | 0.81 | -1.80E-02 | -1.8E-01, 1.4E-01 | 0.82 |
| **HDL (mmol/L)** | 2.50E-01 | -3.8E-01, 8.8E-01 | 0.44 | 1.50E-01 | -2.4E-01, 5.4E-01 | 0.44 | -7.70E-02 | -5.9E-01, 4.3E-01 | 0.76 |
| **Creatinine (per 10 mmol/L)** | -7.30E-02 | -3.1E-01, 1.6E-01 | 0.55 | -3.80E-02 | -1.9E-01, 1.1E-01 | 0.62 | 9.10E-02 | -8.9E-02, 2.7E-01 | 0.32 |
| **Glucose (mmol/L)** | -3.00E-02 | -1.5E-01, 8.6E-02 | 0.61 | -2.50E-02 | -1.0E-01, 5.3E-02 | 0.53 | 5.70E-02 | -3.6E-02, 1.5E-01 | 0.23 |
| **GlycA (mmol/L)** | 5.20E-02 | -1.8, 1.90 | 0.96 | 6.40E-01 | -5.6E-01, 1.8 | 0.29 | 3.40E-01 | -1.1, 1.8 | 0.65 |
| **CRP (mmol/L)** | 5.20E-03 | -3.2E-02, 4.2E-02 | 0.78 | -4.60E-03 | -2.7E-02, 1.8E-02 | 0.69 | -1.00E-02 | -3.8E-02, 1.7E-02 | 0.46 |
| **Frailty index** | -1.8 | -5.7, 2.0 | 0.35 | -7.40E-01 | -3.3, 1.8 | 0.56 | 1.1 | -2.0, 4.3 | 0.49 |
| **eGFR** | -1.1 | -3.4, 1.2 | 0.36 | -4.40E-02 | -1.5, 1.4 | 0.95 | 1.6 | -2.2E-01, 3.4 | 0.086 |
| **FEV1/FVC ratio** | 1.4 | -8.5, 1.1E+01 | 0.78 | 2.9 | -3.0, 8.8 | 0.34 | 9.20E-01 | -6.6, 8.5 | 0.81 |
| **Number of comorbidities** | 8.90E-02 | -5.8E-02, 2.4E-01 | 0.23 | 3.10E-02 | -6.0E-02, 1.2E-01 | 0.5 | -9.00E-02 | -2.0E-01, 2.4E-02 | 0.12 |
