# Supplementary material for: Potential determinants of antibody responses after vaccination against SARS-CoV-2 in older persons: the Doetinchem Cohort Study
Source: Immun Ageing. 2023 Oct 25;20:57. doi: 10.1186/s12979-023-00382-4 (PMC10599057; doi:10.1186/s12979-023-00382-4)
Supplement: Supplementary file 6 — Additional file 6: Table S5. Multivariate correlation of comorbidities and frailty index parameters with the anti-S1 antibody concentrations 1 month after first vaccination dose (T1), second vaccination dose (T2), and the log-fold change during the primary vaccination series with BNT162b2. Statistically significant (P <= 0.05) associations are made bold. [file 12979_2023_382_MOESM6_ESM.docx]

**Table S5:** *Multivariate correlation of comorbidities and frailty index parameters with the anti-S1 antibody concentrations one month after first vaccination dose (T1), second vaccination dose (T2), and the log-fold change during the primary vaccination series with BNT162b2. Statistically significant (P <= 0.05) associations are made bold.*

|  | ***T1: N=853*** | | | ***T2: N=954*** | | | ***Log-fold change: N=791*** | | |
| --- | --- | --- | --- | --- | --- | --- | --- | --- | --- |
|  | **β** | ***95% CI*** | ***P*** | **β** | ***95% CI*** | ***P*** | **β** | ***95% CI*** | ***P*** |
| ***Age*** | **-4.5E-02** | **-7.5E-02, -1.5E-02** | **0.0033** | **-3.0E-02** | **-4.7E-02, -1.2E-02** | **0.0013** | -4.0E-03 | -3.1E-02, 2.3E-02 | 0.77 |
| ***Having any comorbidity*** | **-6.6E-01** | **-1.2E+00, -9.6E-02** | **0.022** |  |  |  |  |  |  |
| ***Arthrosis*** | 2.8E-01 | -1.4E-01, 7.0E-01 | 0.19 |  |  |  |  |  |  |
| ***Asthma*** | -3.3E-01 | -1.8E+00, 1.1E+00 | 0.66 |  |  |  |  |  |  |
| ***BMI*** | -2.8E-01 | -7.6E-01, 2.0E-01 | 0.26 | **3.1E-01** | **2.5E-02, 6.0E-01** | **0.033** | 4.0E-01 | -2.4E-02, 8.2E-01 | 0.064 |
| ***Cardiac catheterization*** | **-1.9E+00** | **-3.2E+00, -6.0E-01** | **0.0044** | -6.3E-02 | -4.3E-01, 3.0E-01 | 0.73 | 7.9E-02 | -4.5E-01, 6.1E-01 | 0.77 |
| ***Cardiovascular disease*** | **1.7E+00** | **5.4E-01, 2.9E+00** | **0.0046** |  |  |  |  |  |  |
| ***Cognition: memory*** ***<= 10^th^ population percentile*** |  |  |  | -5.2E-02 | -5.0E-01, 4.0E-01 | 0.82 | -2.1E-01 | -8.8E-01, 4.7E-01 | 0.55 |
| ***Cognition: speed <= 10^th^ population percentile*** | -7.0E-01 | -1.5E+00, 7.7E-02 | 0.077 | **-5.9E-01** | **-1.0E+00, -1.4E-01** | **0.011** | **7.0E-01** | **3.7E-02, 1.4E+00** | **0.039** |
| ***Feeling downhearted*** | -3.9E-01 | -1.3E+00, 5.6E-01 | 0.41 |  |  |  |  |  |  |
| ***Diabetes*** | -5.7E-01 | -1.3E+00, 1.8E-01 | 0.13 | -3.4E-01 | -8.1E-01, 1.3E-01 | 0.15 | 5.5E-01 | -1.3E-01, 1.2E+00 | 0.11 |
| ***Gastrointestinal disease*** |  |  |  | **-6.5E-01** | **-1.2E+00, -1.0E-01** | **0.02** | 3.2E-01 | -4.7E-01, 1.1E+00 | 0.43 |
| ***Impaired hearing*** | 2.0E-02 | -1.3E+00, 1.3E+00 | 0.98 |  |  |  |  |  |  |
| ***Joint inflammation*** | 3.9E-01 | -2.4E-01, 1.0E+00 | 0.23 |  |  |  |  |  |  |
| ***Lower-back pain*** | -6.0E-01 | -1.2E+00, 3.1E-02 | 0.062 | **-4.1E-01** | **-8.1E-01, -1.5E-02** | **0.042** | 2.0E-02 | -5.7E-01, 6.1E-01 | 0.95 |
| ***Feeling a lack of energy*** | 5.1E-01 | -4.2E-01, 1.4E+00 | 0.28 | 2.3E-01 | -2.9E-01, 7.5E-01 | 0.39 | -5.8E-02 | -8.2E-01, 7.1E-01 | 0.88 |
| ***Osteoporosis*** |  |  |  | **5.2E-01** | **5.7E-02, 9.8E-01** | **0.028** | 3.6E-02 | -6.5E-01, 7.2E-01 | 0.92 |
| ***Physically inactive*** |  |  |  | -9.0E-01 | -1.8E+00, 4.0E-02 | 0.06 | -2.0E-01 | -1.5E+00, 1.1E+00 | 0.76 |
| ***Psoriasis*** | 5.7E-01 | -1.2E-01, 1.3E+00 | 0.1 |  |  |  |  |  |  |
| ***Low spirometry ratio*** | -2.7E-01 | -7.3E-01, 1.9E-01 | 0.24 | -2.6E-01 | -5.2E-01, 3.1E-03 | 0.053 | -1.6E-01 | -5.4E-01, 2.2E-01 | 0.41 |
